# Flotation using sodium dodecyl sulphate and sodium lauroyl isethionate for rapid dewatering of Mg(OH)_2_ radwaste suspensions†

**DOI:** 10.1039/d1ra01222c

**Published:** 2021-05-24

**Authors:** Alexander P. G. Lockwood, Philip Kok Shun, Jeffrey Peakall, Nicholas J. Warren, Thomas Barber, Nabil Basharat, Geoff Randall, Martyn Barnes, David Harbottle, Timothy N. Hunter

**Affiliations:** School of Chemical and Process Engineering, University of Leeds Leeds LS2 9JT UK T.N.Hunter@leeds.ac.uk; School of Earth and Environment, University of Leeds Leeds LS2 9JT UK; Sellafield Ltd Hinton House, Birchwood Park Ave, Birchwood Warrington WA3 6G UK

## Abstract

Mg(OH)_2_ suspensions were floated utilising sodium dodecyl sulphate (SDS) and sodium lauroyl isethionate (SLI) collectors, for rapid dewatering of radwaste suspensions. Freundlich adsorption isotherms were first used to compare the adsorption densities of SDS and SLI on Mg(OH)_2_ surfaces, to determine the maximum monolayer coverage capacity, and were found to be 0.11 μmol m^−2^ at a dosed concentration of 172 μM for SDS and 0.05 μmol m^−2^ at a dosed concentration of 188 μM for SLI. The natural and salt induced coagulation kinetics of Mg(OH)_2_ were examined using static light scattering, where the influence of collector adsorption on particle size distributions was also investigated, to probe potential hydrodynamic limitations of flotation. Particle stabilised foam formation was then characterised using a Bikerman column test, where the dynamic foamability indices (DFIs) of SDS and SLI were determined to be 49 × 10^3^ s L mol^−1^ and 321 × 10^3^ s L mol^−1^ respectively. Flotation performance was measured, and a collection efficiency factor used to compare the solid–liquid separation ability of mixed 2.5 vol% suspensions with SDS or SLI, as well as MIBC frother. Optimal performance aligned with collector concentrations relating to predicted maximum monolayer coverage, and whilst both surfactants were effective, SDS systems performed better than SLI in all metrics. Recoveries of >80% of the Mg(OH)_2_ wastes were achieved, whilst only transferring 35% of the water mass at the optimum SDS dosed concentration of 82 μM, likely due to its denser surface adsorption and minimised lamella water entrainment.

## Abstract 2

Mg(OH)_2_ suspensions were floated utilising sodium dodecyl sulphate (SDS) sodium lauroyl isethionate (SLI) collectors, for rapid dewatering of radwaste suspensions.

## Graphical abstract

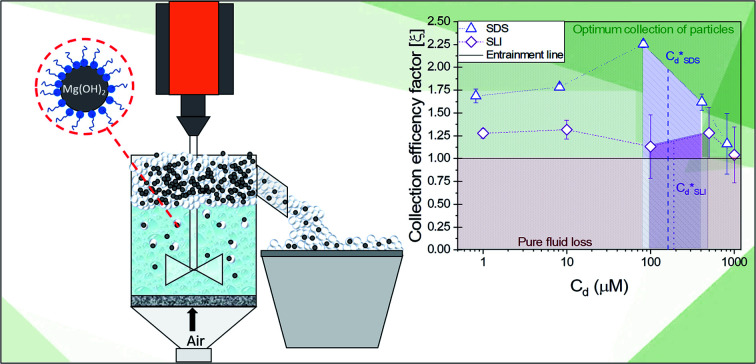

## Introduction

There is a critical need for new flexible and efficient dewatering systems to aid in the decommissioning of legacy nuclear processing facilities worldwide, where the transfer and treatment of multiphase sludge wastes, as part of risk and hazard reduction operations, are a significant area of concern.^1–3^ Challenges from particulate suspensions are ubiquitous in radioactive waste (radwaste) management, in particular as they often contain broad particle size distributions (PSDs) ranging from fine colloidal material to coarse particles and even fuel fragments,^4–7^ along with complex surface chemistries. It is extremely difficult to optimise dewatering processes for the separation of radwaste suspensions, due to their heterogeneity, especially when operations are subject to additional regulatory requirements, such as no moving parts and reduction of secondary waste generation.^8^

A common regulatory driven approach for radwaste dewatering is gravitational thickening, where suspensions are subject to sedimentation, with the less turbid supernatant liquor being pumped back into storage ponds and the separated thickened sludges stored and finally encapsulated.^4,9^ Whist sedimentation is safe and straightforward, it is also slow, with considerable residence times being required in the thickening zones to remove finer particles.^10^ Given the strict timelines that most governments have for the processing and storage of legacy nuclear wastes, optimisation of these dewatering operations is thus a priority for the nuclear industry. To accelerate dewatering, there is current research into the application of polymeric flocculants to enhance suspension zonal settling rates, which have been shown to significantly decrease residence times in thickening operations.^11–15^ However, these processes have a number of drawbacks, in terms of their modification to waste structure and related downstream issues.^1^ In particular, the resultant fractal nature of these polymeric flocs can increase settled bed volumes, which may reduce the solid waste capacity of intermediate level waste containers for final geological disposal.^16,17^

As an alternative to gravitational separation, flotation has received increasing interest from researchers for radwaste separation, as it has been shown to be an extremely rapid dewatering technique, and is already commonly utilised in the minerals, water treatment and paper industries.^18–26^ Previous investigators have studied a range of variables that affect flotation performance, including particle contact angles,^27–29^ bubble size distribution,^25,30^ foam stability,^18,31–34^ suspension/collector concentration,^29,35,36^ collector adsorption density,^23,35,37,38^ collector hydrophobicity,^39–41^ particle coagulation and hydrodynamic consequences of variable PSDs.^15,28,42,43^ Research into flotation of Mg(OH)_2_, a corrosion product of the fuel cladding alloy at Sellafield,^2,6^ has received little interest, but similar mineral particulates have been shown to be effectively separated using flotation facilitated with anionic surfactant collectors, such as alkyl sulphates including sodium dodecyl sulphate (SDS).^19,37,44^

It is thus critical that before the deployment of flotation as a dewatering strategy to separate magnesium hydroxide based radwastes, research is undertaken to establish efficient collectors that are effective at hydrophobising Mg(OH)_2_ particles to allow adsorption onto foam interfaces. Additionally, the objective of flotation for dewatering purposes is not only to successfully remove particles through hydrophobic interactions, but to avoid excess water carry over, which would require secondary waste treatments. Therefore, flotation must be optimised for both particle removal and high dewatering ratios. Additionally, the adsorption dynamics of surfactant collectors onto Mg(OH)_2_ surfaces must be further understood to adapt for varying solid feed concentrations, as it would be expected that monolayer surfactant coverage conditions facilitate optimum recovery of Mg(OH)_2_. Given the impact of collectors and frothers on the foam stability and flotation performance in many mineral systems,^31,33,45^ a wide range of collector agents have been previously investigated. However, sodium lauroyl isethionate (SLI), which is an anionic surfactant consisting of an acid-ester sulphonate head group compared to the SDS sulphate head group,^46^ has not previously been used as a collector in flotation. Thus, it is proposed that an understanding of the differences in collector adsorption, particle coagulation and foamability between two anionic surfactants (SDS and SLI) is key in tailoring a flotation process for effective Mg(OH)_2_ radwaste dewatering.

Here, fine magnesium hydroxide (Mg(OH)_2_) suspensions were used as a radwaste analogue to UK legacy wastes composed of corroded magnesium alloy fuel cladding.^4^ The adsorption of SDS and SLI surfactants onto particle surfaces were characterised using total organic carbon analysis and fitted to heterogeneous Freundlich isotherms, as used in previous studies investigating the adsorption of surfactants onto mineral surfaces.^38,47–49^ The coagulation kinetics of sonicated Mg(OH)_2_ suspensions were also investigated using static light scattering and compared to their surfactant driven aggregation with increasing SDS and SLI concentrations, to determine the collector's effect on particle size distributions. The foamability of particle stabilised suspensions was investigated using the well-established Bikerman column test,^33,41,50^ to calculate the gas retention time and dynamic foamability indices (DFIs) of the particle-surfactant systems, in comparison to non-ionic methyl isobutyl carbinol (MIBC) frother. Finally, the flotation performance of the two surfactant systems was analysed using particle and water mass recovery metrics to calculate residual cell concentrations post-flotation, using a collection efficiency factor incorporating dewatering ratios. The flotation was then holistically analysed by comparing the monolayer surface coverage and resultant hydrophobicity to particle coagulation, foamability and the dewatering efficiency through flotation.

## Experimental

### Materials

Versamag Mg(OH)_2_ (Martin Marietta, US) was used for all experiments. Versamag is a fine, white precipitated powder with a solubility of 6.9 mg L^−1^ at pH 10.1 in water,^51^ and has been previously characterised by Lockwood *et al.*^12^ It is noted that suspensions self-buffer at pH 10–11, due to its increased solubility at lower pHs. Versamag was shown by Lockwood *et al.*^12^*via* electrophoresis to have a surface potential of ∼12 mV and a specific surface area of 8 m^2^ g^−1^ at the self-buffering pH of ∼10.5. Anionic surfactants were selected as collectors as they have been shown to have an affinity to electrostatically adsorb to the surface of positively charged particles.^22,25,44,52^ Solid sodium dodecyl sulphate (SDS) (TOKU-E, ≥99% pure), with a reported critical micelle concentration (CMC) of 8.2 mM,^46^ was dissolved in 0.5 L of Milli-Q™ water to make up a stock solution of 16.4 mM. This solution was stored in cleaned polypropylene containers and diluted with further Milli-Q™ water accordingly for experiments, as used in various previous flotation studies.^19,22,37,38^ Sodium lauroyl isethionate (SLI) (>98% purity), with a recorded CMC of 5.4 mM,^46^ was synthesised and crystallised *via* the methodology outlined by Jeraal *et al.*^46^ It was then dissolved in Milli-Q™ water to make a stock solution of 10 mM, which was stored and sampled for various experiments similarly to the SDS. A stock solution of 100 ppm 4-methyl-2-pentanol (MIBC) (Sigma-Aldrich, 98%, density: 0.802 g mL^−1^) was also utilised in experiments. MIBC is commonly used as a frothing agent for foamability and dispersed air flotation tests.^34,53^ SDS, SLI and MIBC chemical formulas and structures can be found in Table 1.

**Table 1:** List of chemicals used in flotation with their corresponding purposes, chemical formulas and skeletal structures

Chemical	Formula	Purpose	Structure
Methyl isobutyl carbinol (MIBC)	C_6_H_14_O	Frother	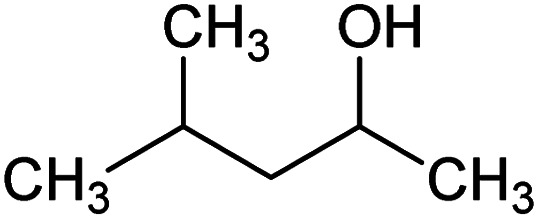
Sodium dodecyl sulphate (SDS)	NaC_12_H_25_SO_4_	Collector	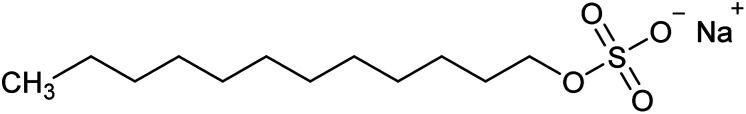
Sodium lauroyl isethionate (SLI)	NaC_14_H_27_SO_5_	Collector	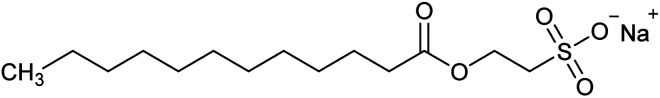

### Particle size analysis

20 mL suspensions were prepared using 2.5 vol% Mg(OH)_2_ suspensions, and were dispersed using an ultrasonic bath (Clifton Sonic) for 20 minutes to breakup any preformed aggregates. The suspensions were then added to a Mastersizer 2000E (Malvern Panalytical Ltd) static light scattering instrument, using a Hydro 2000SM aqueous dispersion cell (external dimensions of 140 × 175 × 390 mm and sample volumes between 50–120 mL). The suspension PSDs were then monitored as a function of time at 900 rpm with KNO_3_ (Sigma Aldrich) background electrolyte at doses of 0, 1 × 10^−3^ and 1 × 10^−2^ M KNO_3_, to observe the effect of salt concentration on Mg(OH)_2_ aggregation formation. KNO_3_ was selected as a simple surrogate for a range of electrolyte ions that are naturally present in the pond wasters, which are unlikely to specifically adsorb onto Mg(OH)_2_ surfaces. The investigation time was intrinsically limited as the Malvern Mastersizer 2000E instrument has an obscuration envelope in which particle size measurements are validly taken. As particles aggregate, the overall number of particles and thus concentration decreases, which reduces obscuration eventually to numbers below the instrument lower limit. To observe the effect of adsorbed collectors on particle aggregation, varying concentrations of collector between 0 and 1000 μM were then added to 2.5 vol% Mg(OH)_2_ suspensions and agitated using a magnetic stirrer for 20 minutes. The Mg(OH)_2_-collector suspensions were then added to the dispersion unit at 900 rpm, to ensure constant shear rates on the aggregated particulates, which could then be compared to the initial aggregation data without collector.

### FBRM measurement

As per the current authors previous methodology for performing FBRM analysis,^12^*in situ* aggregate size determination was conducted using a Lasentec® Focused Beam Reflectance Measurement (FBRM) model PI-14/206 instrument (Mettler-Toledo) in macro mode. The reactor was set-up with the FBRM probe mounted at a 45° angle to the impeller shaft and 10 cm from the reactor vessel base within the mixing zone, to ensure representative flow of suspended particles past the measurement window.^54^ The chord length distribution (CLD) of the system was monitored after allowing a 1 L suspension of 2.5 vol% Mg(OH)_2_ to equilibrate under agitation at 300 rpm for 5 minutes. Chord length number distributions were then computationally translated to volume percentage spherical equivalent diameters, assuming floc sphericity, as outlined by Rhodes^55^ and used in the current authors previous work.^12^

### Collector adsorption onto Mg(OH)_2_

Suspensions of 2.5 vol% Mg(OH)_2_ and varying concentrations of collector ranging from 0.82 μM to 1000 μM, were prepared in centrifuge tubes of 15 mL with the required collector concentration of SDS or SLI diluted from the stock solutions. The suspensions were then agitated using a carousel mixer (Compact Star CS4) for 24 hours, to ensure equilibrium adsorption of the anionic collectors on the Mg(OH)_2_, before being centrifuged at 500 rpm for 4 hours to separate the particulates from the supernatant liquor. The supernatant liquor was then sampled using a needle and syringe through a 0.45 μm syringe filter to ensure no fine suspended material remained in the liquid. Remaining organic carbon concentration was determined using an IL550 Total Organic Carbon (TOC) analyser (Hach-Lange) and was translated to SDS and SLI concentrations using the stoichiometric ratios (see Table 1). Concentrations were quantified by comparing to pure collector solutions as benchmarks, where the difference in collector concentration was used to determine the amount of collector adsorbed onto the Mg(OH)_2_.

Surfactant adsorption was analysed using the Freundlich adsorption isotherm, which is commonly used to measure the adsorption of collectors onto the surface of particles.^47–49^ The linear form is shown in eqn (1), where *q*_e_ is the adsorption density of the collectors onto the Mg(OH)_2_ surface, and in this study was calculated in units of both mg g^−1^ and μmol m^−2^ (by dividing through by the relative molecular mass of the collector (*M*_r_) and the specific surface area (*A*_s_) of the particles, with *A*_s_ = 8 m^2^ g^−1^).^12^*C*_e_ is the equilibrium concentration of collector in the aqueous medium, 1/*n* is the Freundlich constant, and is related to the adsorption energy, while *k*_d_ is the adsorption affinity and essentially relates to the adsorption limit at infinitely small surfactant concentrations.1
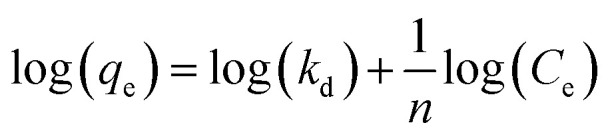


### Foamability tests

Dynamic foam stabilities of Mg(OH)_2_ collector mixtures and MIBC frother were investigated using a fritted glass burette with 19 mm internal diameter and 400 mm height, analogous to the Bikerman column experimental designs implemented by Gupta *et al.*,^34^ Laskowski and Cho^30,33^ and Hunter *et al.*,^18^ MIBC foamability tests were conducted by preparing five 35 mL samples of MIBC at dosages of 2, 10, 25, 50 and 100 ppm with 2.5 vol% Mg(OH)_2_ suspensions without collectors initially to determine a baseline foamability of the MIBC in the electrolyte background provided by the Mg(OH)_2_ semi-solubility. Foamability analysis was performed by combining frother (at 1 ppm) with 2.5 vol% Mg(OH)_2_ suspensions, along with SDS or SLI at various concentrations and superficial gas flow rates from 0 μM^−1^ to 250 mL min^−1^ at fixed 50 mL min^−1^ intervals. The height of the foam layer was recorded after reaching equilibrium, and the airflow was then increased. After the system re-equilibrated, the foam height from the liquid–foam interface was again measured, and the process was repeated until the foam height began to grow non-linearly against flow rate.

Experiments were undertaken with varying dosed concentrations of SDS from 0.82 μM to 9.84 μM and SLI from 1 μM to 40 μM (noting these represented initial added concentrations) and were completed in triplicate. The retention time (*t*_r_), which is often referred to as the Bikerman coefficient,^56,57^ is established from the slope of the linear part of the dependence of the total gas volume in solution and foam, plotted with respect to gas flow rate (for increasing frother or collector concentrations). As the diameter of the Bikerman column is constant, measurements can be reduced to changes in foam height (*H*_f_) and superficial air velocity (*u*) as shown in eqn (2). The dynamic foamability index (DFI) can then be determined using the procedure used by previous authors.^33,34^ The DFI allows comparison of collector and frother foamabilities independent of concentration, for a more robust assessment of flotation performance than retention time alone. The DFI is obtained from the *t*_r_ values as a function of the dosed concentration (*C*_d_) limiting slope, as *C*_d_ approaches 0, shown in eqn (3).2
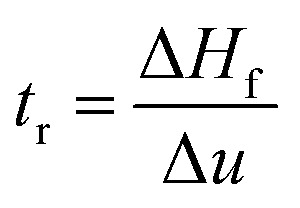
3
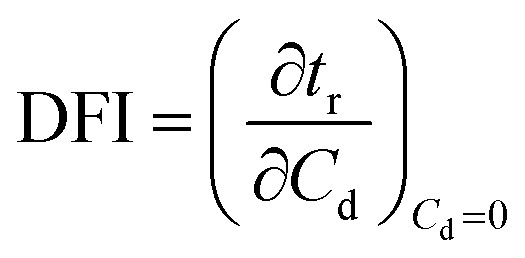


### Floatation experiments

**Fig. 1:**
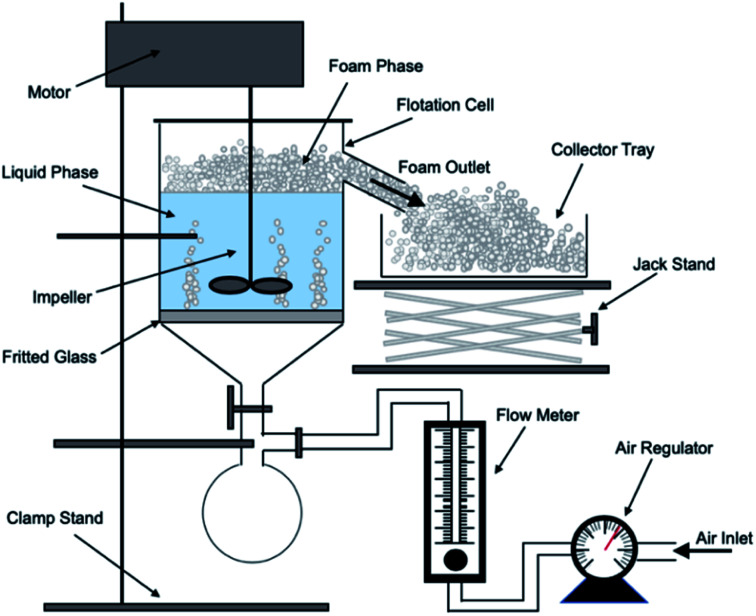
Schematic of batch flotation cell used for dispersed air flotation tests.

A bespoke floatation cell (210 mL, 65 mm ID; Fig. 1) was manufactured with an air inlet and a fritted glass base similar to the designs used by Zhang *et al.*^53^ and Prajitno *et al.*^35^ Here, 12.31 g of Mg(OH)_2_ was added to a measuring cylinder and dosed with 98 μM of MIBC as per this previous work,^35,53^ along with the required dose of SDS, and then made up to 210 mL with Milli-Q™ water. The cell was stirred for 20 minutes at 250 rpm to facilitate adequate adsorption of SDS to Mg(OH)_2_ surfaces. Airflow into the bottom of the cell was set at 0.1 L min^−1^ and the agitator speed was reduced to 100 rpm to minimise turbulence in the cell, preventing bubble disengagement. Froth generated above the air–water interface was collected through the outlet at the top of the vessel, and into an oven for 24 hours to evaporate the water component of the foam, leaving behind the recovered particulates. The recovered solids were then weighed to determine a number of performance indicators.

The Mg(OH)_2_ particle recovery percentage was measured, as shown in eqn (4), where the recovery percentage, *P*_%_, from Mg(OH)_2_ suspensions was calculated using a mass balance approach, as suggested by Zhang *et al.*^53^ It is the percentage of the mass of Mg(OH)_2_ recovered from the initial suspension in the foam phase, where *M*_rp_ is the recovered mass of Mg(OH)_2_ from the foam phase and *M*_Tp_ is the total initial mass of Mg(OH)_2_ in the suspension.4
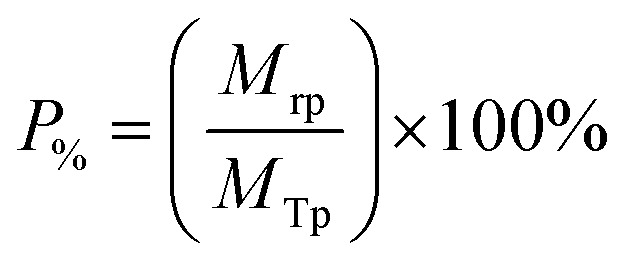


The percentage of fluid remaining in the cell, *W*_%_, was also calculated from the measured mass of the water in the foam phase, *M*_rw_, extracted from the flotation cell. It was obtained differentially from the mass of the aluminium collection container before and after evaporation, and divided by the total initial mass of water in the cell *M*_Tw_, as given in eqn (5).5
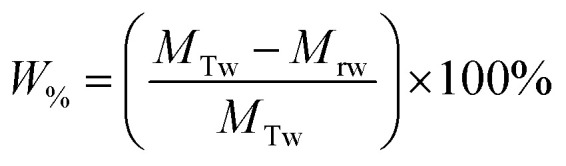


The Mg(OH)_2_ particle concentration remaining in the flotation cell, *C*_%_, can then be determined from mass balance principles, as shown in eqn (6).6
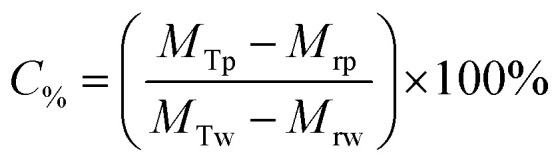


The performance of the collectors were then compared using a collector efficiency factor, *ξ*, shown in eqn (7), which is a ratio of the percentage fraction of the particles-to-fluid recovered from the flotation cell. When *ξ* > 1, there are more Mg(OH)_2_ particles recovered than water by mass, when *ξ* = 1 there is equal particle-fluid extraction (entrainment) and when *ξ* < 1 there is more fluid being extracted than Mg(OH)_2_ particles (indicative of overly wet froths). The collection efficiency factor can then be used to determine the optimum dose of collector to maximise solid–liquid separation.7
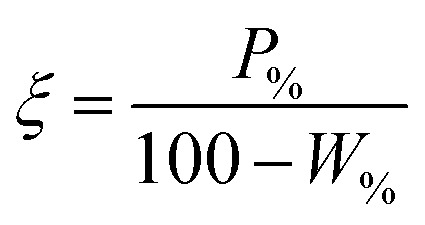


## Results and discussion

**Fig. 2:**
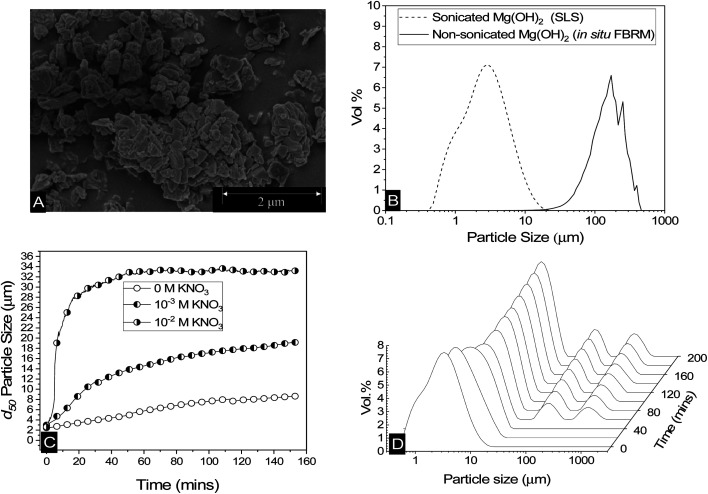
(A) Scanning electron micrograph of dry Mg(OH)_2_ powder. (B) Particle size distributions of sonicated Mg(OH)_2_ dispersions agitated at 900 rpm measured using static light scattering, and non-sonicated agitated at 300 rpm measured *in situ* using focused beam reflectance measurement. (C) Change in the 50^th^ cumulative percentile (*d*_50_) particle size with time of Mg(OH)_2_ dispersions agitated at 900 rpm, along with the addition of 10^−2^ M and 10^−3^ M KNO_3_ at 900 rpm. (D) The change in the volume based (vol%) particle size distribution of Mg(OH)_2_ agitated at 900 rpm with time.

Scanning electron micrographs of Mg(OH)_2_ reveal a structure made up of aggregates of pseudo-hexagonal platelets as shown in Fig. 2A, similar to those reported by Johnson *et al.*^9^ and Maher *et al.*,^7^ and previous work by the current authors.^12^ This fused nanocrystallite platelet arrangement, gives rise to the large relative surface area in terms of that expected from spherical equivalent estimations,^58^ which is an important factor to consider when evaluating the adsorption density of collectors to the Mg(OH)_2_ surface.^52^ The Mg(OH)_2_ specific surface area was investigated previously^12^ using a Brunauer–Emmett–Teller (BET) approach, and was found to be ∼8 m^2^ g^−1^. Whilst this could be considered to be a high specific surface area, for particle agglomerates of micron size, Biggs *et al.*^59^ have observed similar Mg(OH)_2_ material as having a specific surface area of 15.43 m^2^ g^−1^, due to its fractal nature and high internal porosity.^60^ Similar mineral pseudo-hexagonal platelet material, such as aluminium hydroxide, have also been found to have correspondingly high BET surface areas, ranging from 1.5 m^2^ g^−1^, as observed by Adekola *et al.*,^61^ up to 91 m^2^ g^−1^ measured by Rosenqvist.^62^

The low surface potential of magnesium hydroxide (of ∼12 mV, as noted in the methodology^12^) has implications when considering colloidal stability of particulates, suggesting they may naturally aggregate.^63^ Aggregation may affect the accessible surface area in aqueous media, resulting in consequences for adsorption density.^52^ Additionally, increases in particle size distributions (PSDs) may potentially lead to hydrodynamic limitations in the flotation process.^15,28^ Therefore, a series of measurements were performed to understand particle size changes in dispersions. Fig. 2B shows the differences in PSDs of sonicated Mg(OH)_2_, using static light scattering, where the sample was dispersed in a small distribution cell at 900 rpm, with an approximate median particle size (*d*_50_) of 2–3 μm. Also shown in Fig. 2B is a non-sonicated dispersion, measured *in situ*, using the focused beam reflectance measurement (FBRM) technique, which presents a considerably larger range, with comparative *d*_50_ = 149 μm. Whilst these techniques are different (and noting the FBRM represents a volume based conversion from raw chord length distributions^12^) the significant disparities in PSDs indicate that Mg(OH)_2_ suspensions will naturally further aggregate under low shear conditions, due to their low zeta potential.^63^ Previous work by Johnson *et al.*^9^ on the same type of Mg(OH)_2_ also found naturally coagulated aggregates in the range of 150 μm, consistent with the FBRM data.

The change in the 50^th^ cumulative percentile (*d*_50_) particle size with time of Mg(OH)_2_ suspensions is shown in Fig. 2C; these were also sonicated for 20 minutes to break up any preformed aggregates. The post-sonication re-aggregation of the sonicated sample can be observed by the change in *d*_50_ with time when added to the Mastersizer dispersion cell at 900 rpm, along with KNO_3_ electrolyte backgrounds of 10^−3^ M and 10^−2^ M. For all datasets, the *d*_50_ increased with time indicating different degrees of particle aggregation. The role of electrolytes in electric double layer compression, resulting in greater aggregation is well documented in previous literature,^48,64–66^ with the data in Fig. 2C following expected trends, where an increase in KNO_3_ concentration results in a greater degree and rate of aggregation.^65,67^

The impact of aggregation on polydispersity is shown in Fig. 2D in the form of PSDs of sonicated suspensions evolving over time. It is often difficult to completely understand the aggregation process when considering a single number to represent a whole particle size distribution, such as the *d*_50_. Therefore, an insight into the change in particle size dispersity and PSD transition to polymodality can provide important information on the aggregation mechanism. For times up to 40 minutes, there is a gradual shift in the PSD peaks to the right, indicating a gradual size increase (similar to that captured by Fig. 2C in increasing *d*_50_ values). At 40 minutes however, the emergence of two additional peaks in the region of ∼100–1000 μm is evident. The magnitudes of these additional peaks (vol%) increases with time, accompanied by a continuing shift to the right of the initial peak (∼0.1–20 μm). The emergence of the two additional peaks (∼100–1000 μm) does not visibly impact the trajectory of the *d*_50_ size increase with time (in Fig. 2C) as their relative magnitudes are much lower than that of the initial peak, and they also do not lead to significant skewing of *d*_50_ value (highlighting the limitations of using this single value). It is additionally noted that the relatively high shear rate in the instrument cell (900 rpm) may lead to continual aggregate breakage, reducing the development of these larger size peaks. It is also likely why large single macro-aggregate peaks are not observed, as evidenced with the *in situ* FRBM data (as these measurements were gained in a larger 300 rpm low-shear cell).

What is particularly important regarding the emergence of these additional peaks at ∼100–1000 μm at intermediate times, is the inference of the distinctive development of a new aggregation mechanism. The initial peaks (*t* < 40 min) indicate a particle-cluster aggregation mechanism, where the dispersed Mg(OH)_2_ gradually aggregates by a particle wise addition to developing Mg(OH)_2_ clusters. As time elapses and more of these clusters are formed, this results in more probabilistic cluster–cluster macro-aggregation,^68,69^ represented by the additional two peaks after 40 minutes. As time develops, these peaks overlap as can be observed in the 10^−2^ M KNO_3_ system in the ESI Fig. S1,† which displays more advanced aggregation within the time observed, due to the depletion of the electric double layer from the KNO_3_ elctrolytes.^67^ Finally, the PSD reaches an equilibrium, which is a function of the shear rate in the dispersion cell (*i.e.* greater shear reduces equilibrium particle size).^12,17,70–73^

### Collector adsorption and effect on particle aggregation

**Fig. 3:**
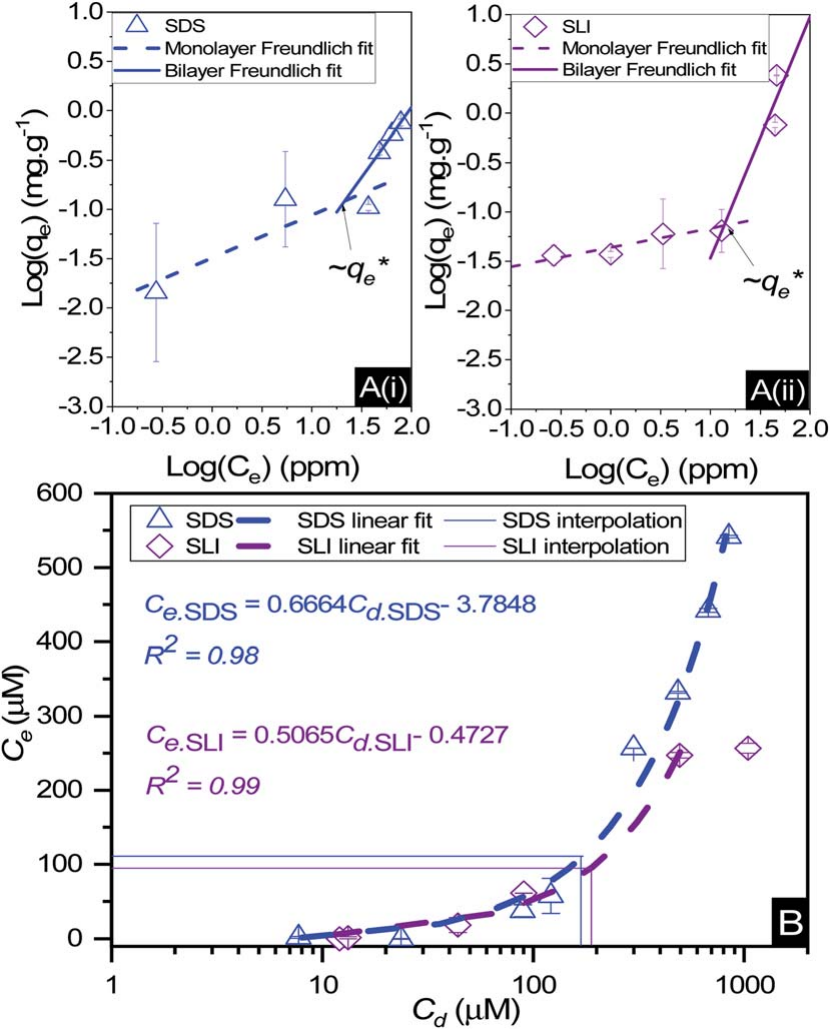
(A) Two region fitted Freundlich adsorption isotherm including both monolayer and bilayer adsorption profiles for (i) sodium dodecyl sulphate and (ii) sodium lauroyl isethionate collectors on Mg(OH)_2_. It is noted that 
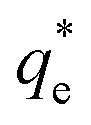
 is the maximum monolayer collector adsorption density (monolayer to bilayer transition point). (B) Calculated equilibrium concentration, *C*_e_, established from the dosed collector concentration (*C*_d_) minus the amount of surfactant adsorbed. The monolayer–bilayer transition concentration is shown for both surfactants by interpolation.

The specific adsorption of SDS and SLI onto Mg(OH)_2_ as a function of concentration is shown in Fig. 3A(i) and (ii) presented as the logarithmic adsorption capacity, log(*q*_e_), plotted against the logarithmic collector equilibrium concentration, log(*C*_e_), for SDS and SLI respectively. The linear form of the Freundlich adsorption isotherm (eqn (1)) was fitted to the data in two clear regions, which were assumed to represent monolayer and bilayer (ad micelle) regions respectively, commonly found with charged surfactant adsorption on solid surfaces.^35,74^ The associated Freundlich adsorption coefficients were extrapolated from the linear fittings and are displayed in Table 2. The intercept of the monolayer and bilayer Freundlich adsorption isotherms represents the collector equilibrium concentration, where the monolayer to bilayer transition is assumed to represent the maximum monolayer coverage adsorption density (denoted 
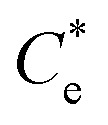
 and 
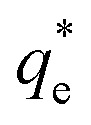
 respectively). The corresponding initial collector dosage concentration representing this transition, 
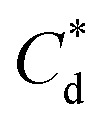
, can be interpolated from the plot of initial dosed collector concentration, *C*_d_, and the collector supernatant equilibrium concentration, *C*_e_, (shown in Fig. 3B). The Freundlich adsorption isotherms have relatively good fits with *R*^2^ values ranging from 0.87 to >0.99, although the fits were notably better for the bilayer adsorption regimes, which is likely due to the lower experimental uncertainty of carbon concentration analysis at higher collector concentrations. A feature of the Freundlich adsorption model is that it is derived by assuming an exponentially decaying adsorption site energy distribution. As the Freundlich constant (1/*n*) that based on adsorption density increases, this represents a greater adsorption intensity on the Mg(OH)_2_ surface occurs with the bilayer.^49^

**Table 2:** Freundlich adsorption isotherm coefficients for sodium dodecyl sulphate (SDS) and sodium lauroyl isethionate (SLI) monolayer and bilayer adsorption profiles determined from linear fittings in Fig. 3A(i) and (ii). Here, *K*_d_ and 1/*n* are the Freundlich coefficients related to the adsorption affinity and intensity respectively, 
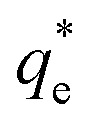
 is the maximum monolayer collector adsorption density (*i.e.* monolayer to bilayer transition point), 
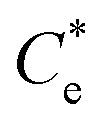
 and 
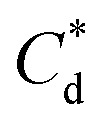
 are the collector supernatant equilibrium and initial dosed concentration respectively at the monolayer to bilayer transition point

Isotherm value	Sodium dodecyl sulphate (SDS)		Sodium lauroyl isethionate (SLI)	
	Monolayer	Bilayer	Monolayer	Bilayer
Freundlich *R*^2^	0.89	0.99	0.87	0.96
*K*_d_ (mg g^−1^)	3.21 × 10^−2^	1.60 × 10^−3^	4.43 × 10^−2^	1.21 × 10^−4^
*K*_d_ (μmol m^−2^)	1.39 × 10^−2^	6.94 × 10^−4^	1.61 × 10^−2^	4.39 × 10^−5^
1/*n*	0.43	1.41	0.2	2.44
*n*	2.30	0.71	5.10	0.41
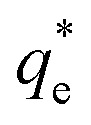 (μmol m^−2^)	∼0.11		∼0.05	
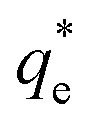 (mg g^−1^)	∼0.24		∼0.14	
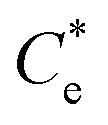 (μM)	∼111		∼95	
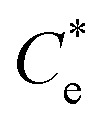 (mg g^−1^)	∼0.03		∼0.03	
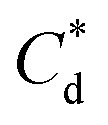 (μM)	∼172		∼188	
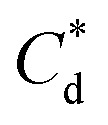 (mg g^−1^)	∼0.05		∼0.03	
Linear *R*^2^	0.98		0.99	

By comparing the monolayer adsorption profiles for SDS and SLI, it is apparent that the adsorption intensity is greater for SDS than SLI, with corresponding 1/*n* values of 0.43 and 0.2 respectively. These 1/*n* values are similar to those observed by Yekeen *et al.*^48^ who investigated the adsorption of SDS onto kaolinite in the presence of Al_2_O_3_ and SiO_2_ nanoparticles. The maximum monolayer adsorption capacities 
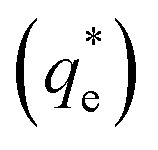
 determined from the intercept of the monolayer and bilayer Freundlich adsorption isotherms of the SDS and SLI are ∼0.11 and ∼0.05 μmol m^−2^ respectively. These values are slightly higher than those observed by Yekeen *et al.*,^48^ who found maximum SDS adsorption onto Al_2_O_3_ nanoparticles to be ∼0.04–0.08 μmol m^−2^ (5.102 mg g^−1^, where *A*_s_ = 230–400 m^2^ g^−1^ ^48^) where it is assumed the Al_2_O_3_ particles are similarly charged to the magnesium hydroxide (although specific chemical affinities may be different).

When comparing SDS to SLI, it is noted that due to the acid-ester sulphonate head group, SLI has a lower hydrophilic head electron cloud density than SDS, which is due to the difference in chemical structure (see Table 1). The reduced electron density is due to SLI having an additional ethyl chain between the S and O (ergo isethionate functional group) unlike in the SDS (which has a sulphate head group). The increased hydrophilic head group size will act in conjunction with the longer hydrophobic chain length (which may induce steric hindrance) to overall reduce the maximum monolayer adsorption density compared to SDS, as observed in Fig. 3A(i) and (ii).

When considering the bilayer adsorption regimes, a critical characteristic is the adsorption intensity is greater than the monolayer regime, but contrary to the monolayer regime, the adsorption is noticeably greater for SLI than SDS in the bilayer region, likely due to its longer carbon chain leading to a greater degree of hydrophobicity. The sudden increase in adsorption intensity is caused from the formation of surface aggregates of the monolayer (*hemimicelle*) that are derived from the lateral interaction of hydrocarbon chains. This lateral attraction generates an additional driving force to superimpose the existing electrostatic attraction, causing a sharp increase in adsorption, due to the reduction in free energy that occurs by reducing the degree of H_2_O dipole orientation around exposed collector hydrophobic tails, thus forming a bilayer at a greater intensity than the initial monolayer.^49,63,75–79^

**Fig. 4:**
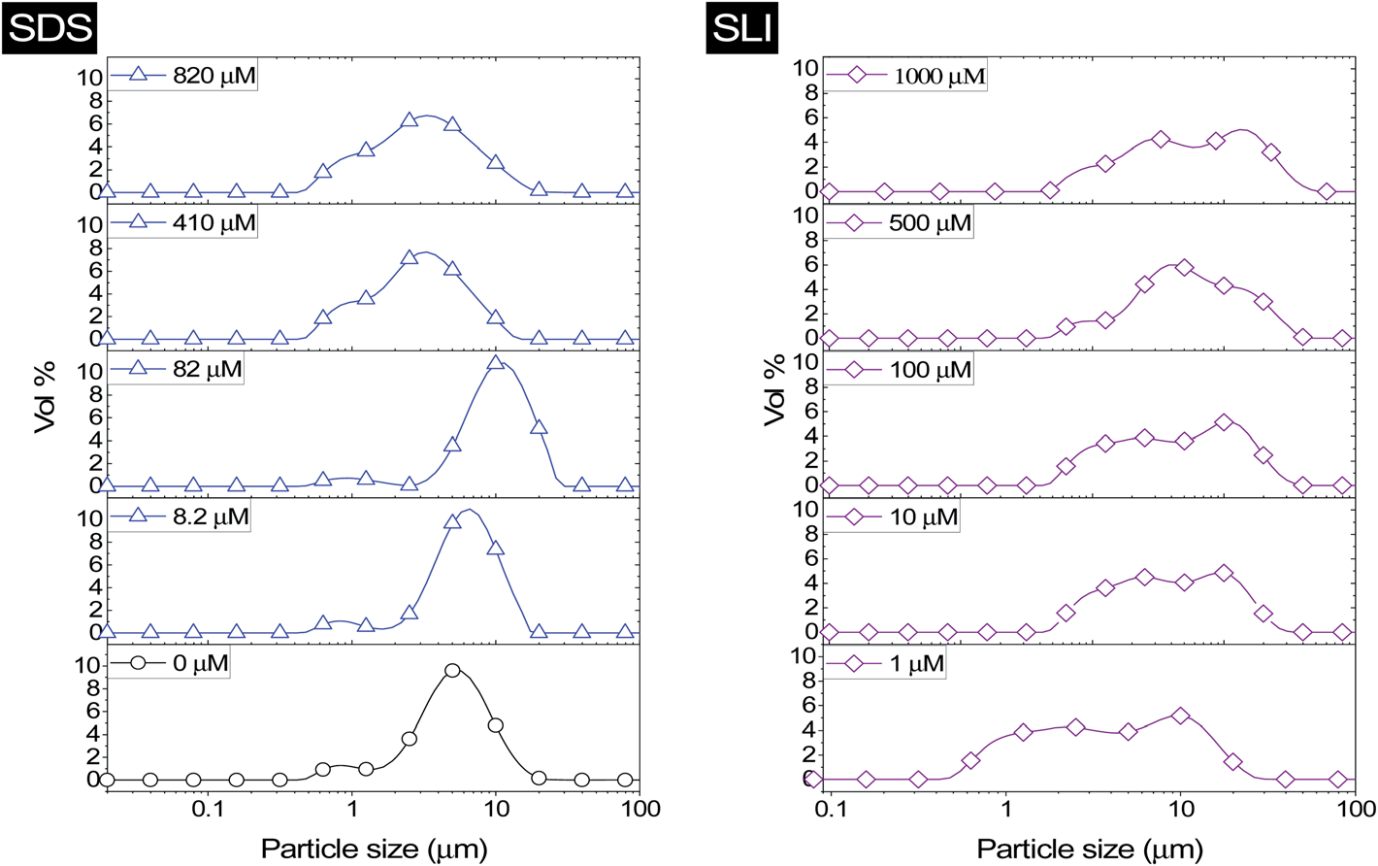
Particle size distributions for Mg(OH)_2_ suspensions sonicated for 20 minutes, dosed with varied concentrations of sodium dodecyl sulphate (SDS) and sodium lauroyl isethionate (SLI) between 0–820 μM and 0–1000 μM respectively and stirred for 20 minutes, before analysis with static light scattering using a 900 rpm flow cell.

The effect of surfactants on the aggregation of sonicated Mg(OH)_2_ dispersions is presented as the volume based PSDs for Mg(OH)_2_ suspensions (initially sonicated to break up any preformed aggregates) dosed with collector concentrations ranging from 0–820 μM for SDS and 0–1000 μM for SLI (below both SDS and SLI CMCs) in Fig. 4. There are distinctive changes in the PSDs with varying doses, most noticeably at concentrations above the 
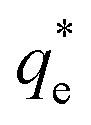
 max adsorption density values (determined in Fig. 3A for both systems, at ∼172 μM and ∼188 μM for SDS and SLI respectively).

For 0 μM collector dose, the PSD may be considered as pseudo-bimodal, with a small peak at 0.3–2 μm of magnitude ∼2 vol% and a major peak at 2–20 μm of magnitude ∼8 vol%. The smaller peak is likely the size of the primary particles, which were determined by Lockwood *et al.*^12^ to be ∼0.3 μm *via* SLS, whereas the larger peak is likely the equilibrium particle size achieved by sonication (with some initial particle-cluster aggregation as discussed in Fig. 2C and D). When considering the SDS systems, when 
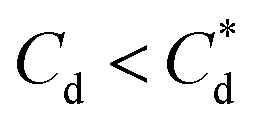
, there is a visible shift of the larger peak to the left accompanied by gradual peak broadening, indicating an increase in particle size from enhanced aggregation conditions. When 
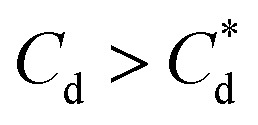
, the particle size distribution reduces again, back towards the 0 μM distribution (although, with a broader monomodal peak). Potentially, the high concentration of SDS may have resulted in some aggregation of the primary particles in the smaller peak size range (0.3–2 μm), but it should be noted that the span of the PSD does not explicitly show any increase, which may indicate that the particle size maxima are shear rate dependent. Alternatively, the greater surface coverage of SDS may stabilise smaller nucleation clusters similar to how surfactants stabilise nanoparticle systems,^32,80,81^ preventing the initial cluster–cluster aggregation processes from developing into those observed in Fig. 2D at 40 min. The SLI system shows similar trends to SDS, however, there is greater PSD dispersity at 
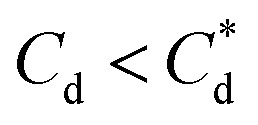
 and the overlap of the primary particle size range (0.3–2 μm) and the main peak (2–20 μm) is visible as an emerging peak at ∼2–8 μm above 100 μM SLI doses (*i.e.*
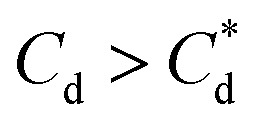
). The SLI also displays a less clear trend of particle size reduction in the bilayer region, suggesting a reduced dispersant property for the SLI in comparison to the SDS at high concentrations.

Prajitno *et al.*^35^ investigated the effect of ethylhexadecyldimethylammonium bromide and cetylpyridinium chloride collectors adsorbed onto clinoptilolite, in terms of suspension dispersity using SLS. Whilst Prajitno *et al.*^35^ observed no significant shift in the clinoptilolite PSD *d*_50_ with collector adsorption, increased polydispersity was observed similar to the SDS and SLI systems in this work. Similar to the SDS and SLI, the hydrophilic head group (in this case cationic) of the surfactants investigated by Prajitno *et al.*^35^ adsorbed onto the anionic clinoptilolite particle surface, resulting in the clinoptilolite particles having greater hydrophobicity at monolayer coverage (similar to SDS and SLI in Fig. 3A). The greater hydrophobicity resulted in a greater surface energy in the water environment, reducing stability leading to aggregation, although not to a significant enough extent to facilitate considerable flocculation.^35^ Like this system, the adsorption of SDS and SLI should not present any significant issues regarding the suspension's dispersion stability,^35^ indicating that the PSD of the suspension in flotation is likely best represented by the *in situ* measurements taken in Fig. 2B.

### Foamability

**Fig. 5:**
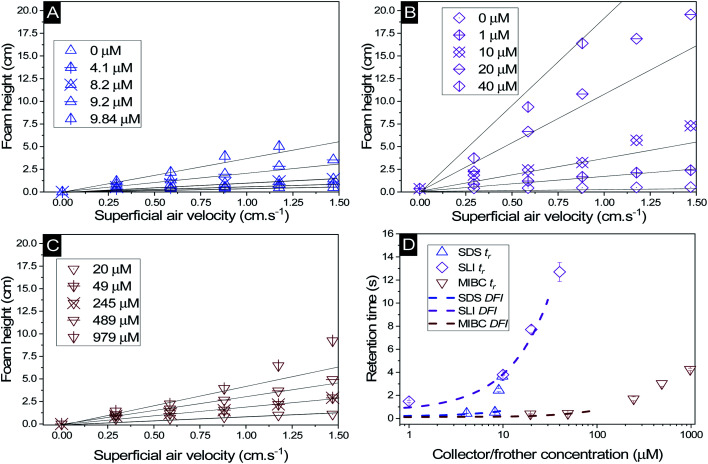
Change in foam height with superficial air velocity for (A) SDS, (B) SLI, and (C) MIBC, all with 2.5 vol% Mg(OH)_2_ suspensions. (D) The retention time was calculated using eqn (2), with varying collector or frother concentration. Solid lines in (A) to (C) represent linear trendlines to determine the gas retention time (*t*_r_). Dashed lines in (D) represent the linear fit of the dynamic foam stability index (DFI) as per eqn (3).

The change in foam height *versus* surfactant or frother concentration found in the Bikerman column tests for SDS, SLI and MIBC respectively (all in 2.5 vol% magnesium hydroxide suspensions) are shown in Fig. 5A–C. For each of these investigations, the volume of foam increases linearly at lower air flowrates, before entering an unstable non-linear region, indicative of airflow turbulence in the burette.^18,30,34,57,82^ Also, for the SDS and SLI collector systems, the maximum experimental concentrations presented represent the transition point before the foam became significantly unstable (and no accurate measurements at higher concentrations were possible). The upper relative concentrations of SDS and SLI are within the regions for monolayer surfactant coverage (see Fig. 3) where significant hydrophobisation of particles is assumed to occur. It was observed visually that Mg(OH)_2_ particles in this concentration region for both surfactants were transported into the foam phase, causing a heterogeneous froth, where less particle dense regions collapsed, forming cavities in the foam structure preventing further foam volume expansion (see ESI Fig. S2†). The SDS had a lower boundary concentration for this effect than SLI, with maximum experimental concentrations of 9.84 μM and 40 μM respectively (see Table 3). This difference is likely related to the greater adsorption intensity of SDS onto Mg(OH)_2_ surfaces at lower concentrations (again, see Fig. 3). It is also noted that no such effects were evident with the uncharged MIBC frother, and it is assumed to not have any considerable interaction with mineral cationic surfaces. Therefore, MIBC tests were conducted over a much larger concentration range.

**Table 3:** Maximum surfactant or frother concentration measurable in Bickerman foam tests, due to particle overstabilisation preventing foam development, and corresponding dynamic foamability index (DFI), calculated using eqn (3)

System	Max conc. (μM)	DFI (s L mol^−1^)
Sodium dodecyl sulphate	9.84	49 × 10^3^
Sodium lauroyl isethionate	40	321 × 10^3^
Methyl isobutyl carbinol	979	6 × 10^3^

The retention time of the gas in the foam phase, with varying concentrations of each collector or frother (calculated using eqn (2)) was measured and is presented in Fig. 5D. While the retention time does increase with surfactant concentration for all systems, it does not increase at the same rate, due to the impact of enhanced particle-stabilisation effects in the SDS system (with respect to the SLI-particle system) which complicates the use of retention time as a marker for foam stability in these mixed surfactant-particle cases. The MIBC system achieves greater retention times than the SDS-particle system at high concentrations, but again, this is likely from the concentration limits imposed by the additional foam-particle stability. Indeed, it is noted that in general, MIBC is used as an active frother that promotes stable foams over relatively short periods of time, allowing for high degrees of mineral separation, without long lasting froths that hinder breakdown in onward treatments.^83^

To compare the foamability with a measure independent of concentration, the dynamic foamability indices (DFI) were calculated using eqn (3) and are displayed in Table 3. The collector-particle systems displayed DFIs greater than that of the frother system, where SDS and SLI have DFIs of 49 × 10^3^ and 321 × 10^3^ s L mol^−1^ respectively. The MIBC frother displayed a DFI of 6 × 10^3^ s L mol^−1^, which is lower than previous work done by Gupta *et al.*,^34^ Melo and Laskowski^30^ and Laskowski *et al.*,^33^ who found DFIs of MIBC in the range of 34–37 × 10^3^ s L mol^−1^. However, in the study by Melo and Laskowski,^30^ they also investigated the effect of brine on the DFI of MIBC and found a much lower value of 3.9 × 10^3^ s L mol^−1^. This difference indicates that ion effects on frother activity may significantly impact the DFI of MIBC, and is important in the current systems because of the semi-solubility of Mg(OH)_2_, which at 2.5 vol%, possess an ionic strength sufficient to alter the pH of suspensions to >10–10.5.^12^ In terms of the surfactant systems, a previous study with SDS by Khoshdast *et al.*,^50^ found the DFI to be 92 × 10^3^ s L mol^−1^, and so similar to the value observed in this work (although, any comparisons must be made with caution, owing to the complication of SDS interactions with the Mg(OH)_2_ particles in the present case). As the DFI for SLI solutions (with or without particles) has not been recorded previously in literature (to the authors' knowledge) additional Bickerman column tests were completed with SLI only solutions (see ESI Fig. S3A and B†) where the DFI was calculated to be 324 × 10^3^ s L mol^−1^, and so very similar to the particle stabilised SLI system in Table 3.

The role of particles in foam stabilisation has been extensively researched, where Hunter *et al.*^18,24^ suggested that for strongly hydrophobic particles with contact angles approaching 90°, particles act as steric barrier to bubble coalescence due to their high particle-interface attachment energy. However, particles that are weakly hydrophobic may also stabilise foams through retarding film drainage in the lamella *via* film stratification,^25,40,84–86^ which is more important for dynamic wet foams found in flotation operations, and implies that they have greater water retention at their equilibrium lamella fluid drainage.^18^ Increased water retention is an important factor in flotation as it may lead to increased water carry-over reducing solid–liquid separation efficiencies. Given the greater DFI for SLI, which has lower collector monolayer adsorption densities, increased stabilisation *via* film drainage retardation may entrain greater amounts of water with less particles, potentially lowering the collection efficiency factor for the SLI system. The greater foam volume generated by SLI compared to SDS, combined with the lower adsorption density of the SLI onto the Mg(OH)_2_ particles, indicates that there is a greater water content in the foam. Combined with the potential for particle drainage or lower collection due to the comparatively subpar hydrophobisation, SLI may result in inferior flotation conditions compared to the SDS system.^18,19,22,23,31,33,35,38^

### Flotation performance

**Fig. 6:**
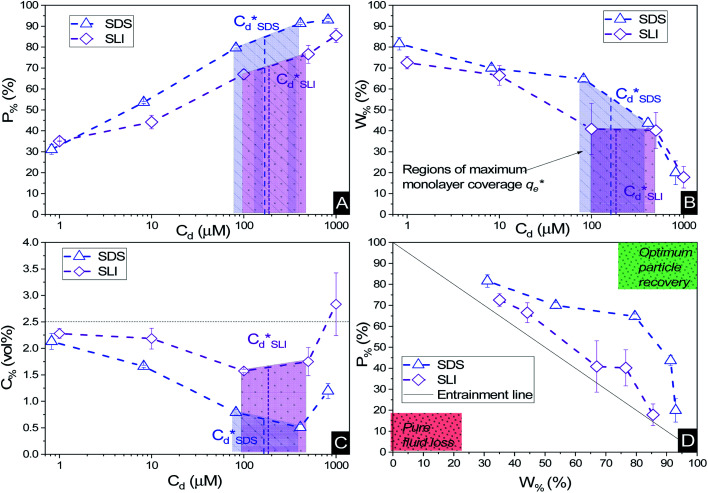
The flotation performance with increasing collector concentration for 2.5 vol% suspensions, as a measure of (A) mass percentage (*P*_%_) of Mg(OH)_2_ particles recovered, (B) mass percentage of water (*W*_%_) remaining in the cell, and (C) the residual Mg(OH)_2_ concentration (*C*_%_) in the flotation cell post flotation. (D) The corresponding mass percentage of water recovered with increasing mass percentage of Mg(OH)_2_ particles recovered. Connecting lines are a visual guide.

Particle recovery as a mass percentage of the total Mg(OH)_2_ particles initially in the flotation cell as a function of collector concentration of SDS and SLI is presented in Fig. 6A. As the collector concentration increases, there is a clear increase in Mg(OH)_2_ recovery from the flotation cell using both collectors, which plateaued at 93% and 86% Mg(OH)_2_ recovery for SDS and SLI respectively. Excluding the first recoded point at ∼1 μM collector concentration, the SDS significantly outperforms the SLI in regards to Mg(OH)_2_ mass recovery. Fig. 6B compares the mass of water remaining in the flotation cell as a function of collector concentration for SDS and SLI. Much like when considering the mass of Mg(OH)_2_ recovered from the flotation cell, SDS outperformed the SLI again, with the region of greatest separation in performance located at the predicted region (data points enclosing 172–188 μM) for maximum monolayer coverage discerned from Fig. 3A and B. The combination of the mass of particles and water recovered from the flotation cell can be used to calculate the residual cell concentration from eqn (6), as shown in Fig. 6C. Consistent with the previous performance measures, again the SDS considerably outperforms the SLI system, achieving the lowest cell volumetric concentrations of 0.5 vol% and 1.6 vol% respectively (from initial suspension concentration of 2.5 vol%). Once again, these optimum performances lay in the maximum monolayer coverage regions (calculated from the adsorption isotherms) as highlighted in Fig. 6A–C.

For SLI, the residual cell concentration post flotation in the bilayer adsorption regime increased, likely due to the increase in fluid loss intensity shown in Fig. 6B when 
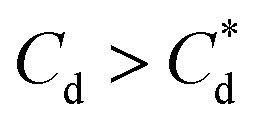
. For greater insight into performance, the fluid loss from the cell and particle recovery were plotted with respect to each other, along with an equal entrainment line (*P*_%_ = *W*_%_) as shown in Fig. 6D. Values above the entrainment line represent a higher ratio of the mass percentages of particle recovery to water remaining in the flotation cell post flotation, *i.e.* greater collection efficiency. Conversely, values below this line represent a greater amount of water being recovered than particles on a mass basis. All data points for SDS and SLI lay above the entrainment line showing at all concentrations of collector dose there is successful particle-fluid separation to some degree, while the heightened performance of SDS is clearly evident across the concentration range.

**Fig. 7:**
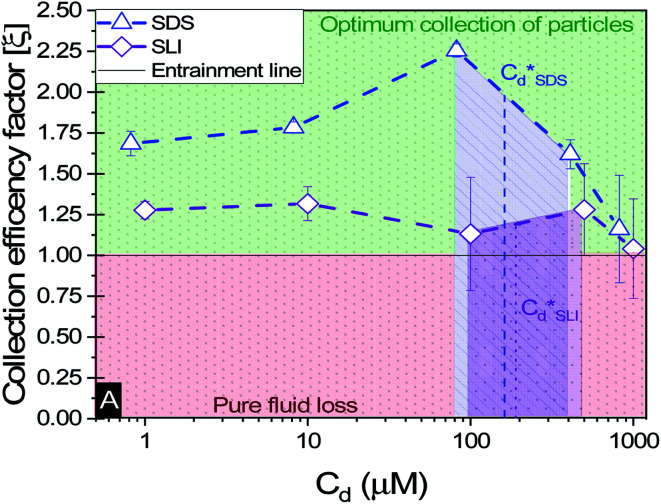
The effect of collector concentration on the collection efficiency factor in eqn (7), displayed with an equal entrainment line. Values above the line represent a greater proportion of particles being recovered and below the line represent greater fluid recovery.

A synthesis of the data from Fig. 6A–D is shown in Fig. 7, allowing scrutiny of collection efficiency as a function of collector concentration, and by extension, the adsorption data shown in Fig. 3A, for a holistic analysis of flotation performance. The ratio of the mass percentage of Mg(OH)_2_ particles and water recovered is displayed as the collection efficiency factor, *ξ*, as per eqn (7). The greater the value of *ξ*, the more efficient the particle separation from water in the flotation cell, where values below 1 (the entrainment line) represent a greater recovery of water from the Mg(OH)_2_ suspension. The maximum *ξ* values coincide with maximum monolayer coverage regions calculated from Fig. 3A. Beyond this point, 
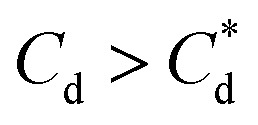
, the collection efficiency tends back towards the entrainment line.

**Fig. 8:**
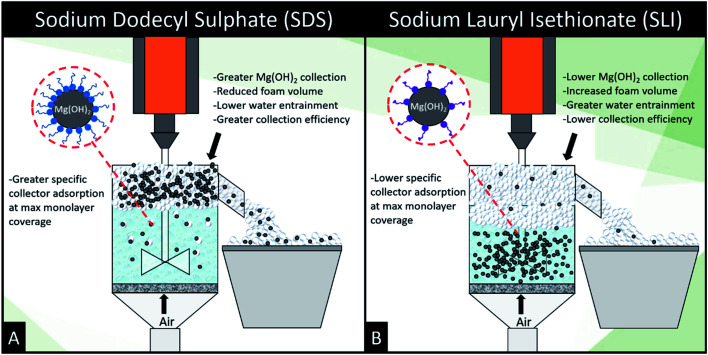
Schematic illustrating the mechanistic differences between (A) sodium dodecyl sulphate and (B) sodium lauroyl isethionate collector flotation systems.

As the maximum monolayer coverage concentration, 
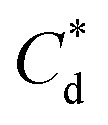
, lies between data points, because of the low data resolution from the logarithmic concentration sweep, the performance factors of the highest performing collector concentrations recorded (by *ξ*) are shown in Table 4. Here one can assume that monolayer coverage is achieved, as the recorded adsorption capacities for these concentrations of SDS and SLI are 96% and 98% respectively of the maximum adsorption capacity 
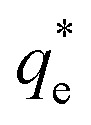
. SDS outperforms SLI at every measure, with greater proportion of Mg(OH)_2_ particles recovered, a greater proportion of water retained in the flotation cell and a lower residual concentration of Mg(OH)_2_ in the flotation cell. Not only does SDS perform better than the SLI by flotation performance metrics, but it is also more efficient on a molecular basis, requiring a lower concentration to achieve a greater collector adsorption density on the surface of the particles to achieve this superior performance. The comparison of flotation performance is illustrated in Fig. 8A and B for SDS and SLI respectively summarising each of the metrics in Table 4.

**Table 4:** Optimum performance data for SDS and SLI, based on their collection efficiency factor (*ξ*). Given is their corresponding mass percentage recovery (*P*_%_), the mass percentage of water remaining in the flotation cell (*W*_%_), the residual flotation cell concentration (*C*_%_), the corresponding surface adsorption density (*q*_e_, from Fig. 3A) and the percentage of maximum capacity adsorption 
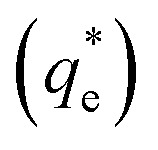
 onto the Mg(OH)_2_ particles

Collector	*C*_d_ (μM)	*ξ*	*P*_%_ (%)	*W*_%_ (%)	*C*_%_ (vol%)	*q*_e_ (μmol m^−2^)	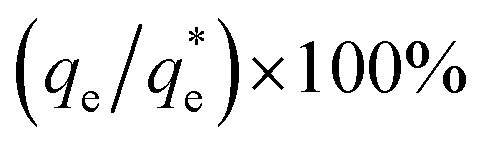
SDS	82	2.3	80	64.7	0.8	0.1	96
SLI	100	1.1	67	40.8	1.6	0.049	98

The importance of collector surface adsorption density and foamability on flotation performance are demonstrated clearly in the flotation data. The adsorption density of collectors on the Mg(OH)_2_ aggregates is vital to increasing the surface hydrophobicity facilitating bubble attachment.^22,23,35,38,53^ SDS generated the greatest particle recovery in this investigation, forming very stable foams with lower water content than SLI (which displayed a significantly greater DFI in Table 3). This increased foamability of the SLI which captured more water in the lamella than the SDS system, and also recovered less particles due to the lower degree of particle hydrophobisation.^87^

The role of collector hydrophobisation in flotation performance is highlighted further in the bilayer adsorption regime. At these concentrations, 
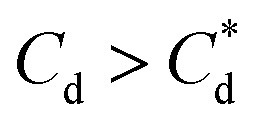
, “*hemimicelles*” begin to form,^49,75,76^ where the second layer of collectors orientate their hydrophobic tails towards the corona of hydrophobic tails in the previously formed monolayer. This lowers the surface energy by reducing the surface area of hydrophobic tails exposed to water dipoles, meaning the bilayer adsorption is entropically driven.^44^ As the polar head groups of the collectors are now facing out into the water, the particles hydrophobicity is decreased, which prohibits bubble attachment as the bilayer coverage increases. The effects of this increase in wettability are observed in the flotation efficiency analysis in Fig. 7, as the collection efficiency factors tend towards 1 when 
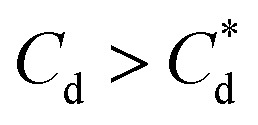
. However, as observed, the collection efficiency factors do not drop below 1 at any concentration, which is likely due to the homogeneous distribution of Mg(OH)_2_ in the well mixed flotation cell. Here, entrained fluid in the foam lamella also has a heterogeneous concentration distribution of Mg(OH)_2_. This means that even in the event of bubble coalescence and lamella drainage, the concentration does not change unless particles are successfully hydrophobically attached to air water interfaces in the foam, where the degree of air water interface adsorption decreases as the bilayer coverage increases, thus *ξ* approaches 1.^18,24,25,84^

An important observation is that at 96% maximum monolayer adsorption capacity 
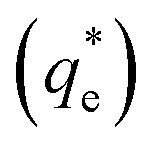
, SDS removed 80% of particles, meaning that 20% of particles remained in the flotation cell. For industrial applications, it would be likely that a second flotation cell operating in series would be required. At higher concentrations further particles were removed, but the efficiency analysis indicates this was through entrainment rather than hydrophobic separation. Previous work into the effect of the hydrophobic tail lengths in carboxylate soap collectors found that increasing the hydrophobic tail length increased the recovery yield in ion flotation.^39^ However, in the present case, the increase in chain length of the SLI did not enhance flotation, largely due to the reduced adsorption density from the larger headgroup as shown in the surfactant-particle adsorption density data in Table 2. Also, increasing the hydrophobicity of particles has a trade off with foam stability. Studies have found that particles with contact angles of ∼70° are optimal for flotation, as greater hydrophobicity particles (contact angles of >90°) have been shown to destabilise, dry and collapse foams due to the increased film drainage.^18,25^ Nevertheless, the low level of aggregation evident (*e.g.*Fig. 5) would suggest such high contact angles are not achieved through either SDS or SLI adsorption.

The relatively fine particle size distributions may additionally suggest a reduced flotation performance of the Mg(OH)_2_, because of the hydrodynamic limitations. Studies into the effect of fine and coarse particles on flotation have found that particles which are too small will lack the inertia required to overcome slipstreams created by the bubbles in turbulent foaming conditions for successful interactions at the air–water interface for particle-bubble attachment.^15,28,88^ While the magnesium hydroxide does aggregate to varying degrees (see Fig. 2) the majority of particles in well mixed conditions are <50–100 μm, with in particular, a high degree of <10 μm fines that will be in the low inertial region. Conversely, aggregates that are too large, may detach from bubbles too easily, because of the greater gravitational forces acting upon them against the buoyancy of rising bubbles.^28^ Although probably less of an issue in the current system, this is why the minerals industry has very high energy requirements for communition of coarse particles to meet the operational envelope of subsequent froth flotation separation stages which is typically estimated as 10–150 μm.^28^ The drive for smarter processing to increase recovery yields and decrease water use has driven innovation in this area to the development collectors, which have the dual functionality of flocculation agents and hydrophobic surface modifiers (usually copolymers).^15,16,43^ However, the current stimuli triggers required to facilitate the switch between hydrophobic and hydrophilic behaviour is problematic for economical/process chemistry concerns, and as a result there is a drive for the development of dual flocculation-collector agents which circumvent the need for stimuli changes.^16^

## Conclusion

The aim of this work was to investigate the application of anionic surfactants, sodium dodecyl sulphate (SDS) and sodium lauroyl isethionate (SLI), as collector agents to dewater Mg(OH)_2_ based radioactive waste suspensions using dispersed air flotation (in the presence of a methyl isobutyl carbinol frothing agent). SDS was found to have a greater adsorption intensity and density in the monolayer regime, with a surface concentration double that of SLI. During bilayer formation, due to the greater surface energy associated with the long hydrophobic chain length, SLI demonstrated a greater adsorption intensity which was entropically driven. Upon sonication, Mg(OH)_2_ was found to readily aggregate, due to its low surface potential in a static light scattering kinetic study, where aggregation was further enhanced in the presence of KNO_3_ salt from reduction of the electrical double layer. The influence of SDS and SLI on the Mg(OH)_2_ particle size distribution (PSD) as a function of collector concentration was investigated and compared to the baseline coagulation study and found to be negligible. An interrogation of the Mg(OH)_2_ particle stabilised foam dynamics using a Bikerman column test showed that the SLI system dynamic foamability index (DFI) was greater than the SDS system. Flotation performance was analysed using a batch flotation cell. Whilst mass and water recovery both increased with increasing collector doses, SDS outperformed SLI as a collector with superior Mg(OH)_2_ recovery and the highest collection efficiency factors. The optimum recovery conditions for both SDS and SLI aligned with the maximum monolayer adsorption density collector concentrations and decreased back to entrainment concentrations in the bilayer regime, which was associated to the decreased hydrophobicity from hemi-micelle formation decreasing the particle surface energy. At the optimal conditions for particle-liquid separation with SDS, 80% of particles were recovered highlighting that flotation is a viable, rapid technique for the dewatering of legacy nuclear wastes.

## Nomenclature

### Scripts

*A*_s_
: Specific surface area, m^2^ g^−1^
*C*_%_
: Mg(OH)_2_ concentration remaining in the flotation cell, vol%
*C*_d_
: Initial supernatant collector concentration, μM
*C*_e_
: Equilibrium supernatant collector concentration, μM
DFI: Dynamic foamability index, s L mol^−1^
*H*_f_
: Foam height, cm
*k*_f_
: Freundlich constant related to the adsorption capacity, mg g^−1^
*M*_r_
: Relative molecular mass, g μmol^−1^
*M*_rp_
: Mass of recovered particles in the collector tray, g
*M*_rw_
: Mass of recovered water in the collector tray, g
*M*_Tp_
: Mass of total particles initially in the flotation cell, g
*M*_Tw_
: Mass of total water initially in the flotation cell, g
*n*
: Freundlich adsorption intensity-based coefficient, —
*P*_%_
: Mass% of Mg(OH)_2_ recovered from the floatation cell, %
*q*_e_
: Collector adsorption density on Mg(OH)_2_ surfaces, mg g^−1^/μmol m^−2^
*t*_r_
: Retention time, s
*u*
: Superficial gas velocity, cm s^−1^
*W*_%_
: Mass% of water remaining in the flotation cell, %
*ξ*
: Collector efficiency factor, —

### Superscripts

*: At maximum monolayer coverage onto Mg(OH)_2_ surface

## Conflicts of interest

The authors declare no competing financial interest.

## Supplementary Material

RA-011-D1RA01222C-s001
