# Supplementary material for: Flotation using sodium dodecyl sulphate and sodium lauroyl isethionate for rapid dewatering of Mg(OH)2 radwaste suspensions
Source: RSC Adv. 2021 May 24;11(30):18661–75. doi: 10.1039/d1ra01222c (PMC9033463; doi:10.1039/d1ra01222c)
Supplement: RA-011-D1RA01222C-s001 [file RA-011-D1RA01222C-s001.pdf]

Electronic Supplementary Material (ESI) for RSC Advances.
This journal is © The Royal Society of Chemistry 2021

Electronic Supplementary Information (ESI)

Flotation using sodium dodecyl sulphate and sodium lauroyl isethionate for rapid dewatering of $Mg(OH)_2$ radwaste suspensions

*Alexander P. G. Lockwood [a] *, Philip Kok Shun[a], Jeffrey Peakall[b], Nicholas J. Warren[a], Thomas Barber[a], Nabil Basharat[a], Geoff Randall[c], Martyn Barnes[c], David Harbottle[a] and Timothy N. Hunter[a,] **

[a]School of Chemical and Process Engineering, University of Leeds, Leeds LS2 9JT, UK.

[b]School of Earth and Environment, University of Leeds, Leeds LS2 9JT, UK.

[c]Sellafield Ltd, Hinton House, Birchwood Park Ave, Birchwood, Warrington WA3 6G, UK.

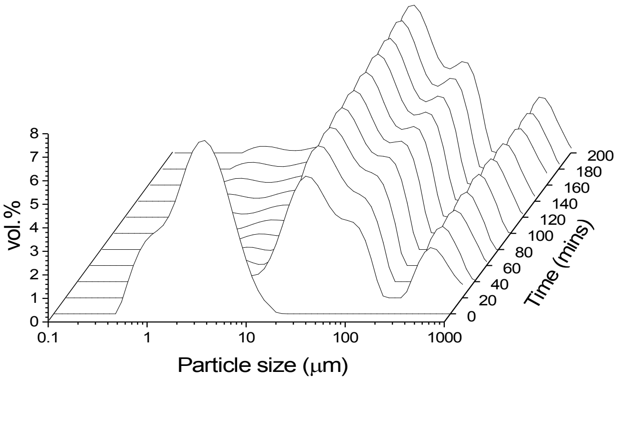

Figure S1: The change in the volume based (vol%) particle size distribution of $Mg(OH)_2$ agitated at 900 rpm and dosed with 10^{-2} M KNO_3 with time.

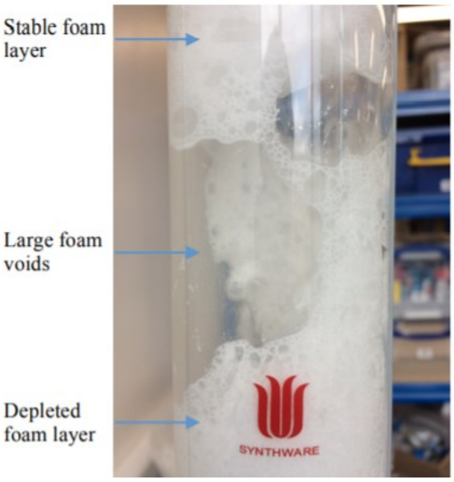

Figure S2: Photograph of Bikerman column during foamability tests, showing stabilised collapsed foam layers preventing further foamability readings.

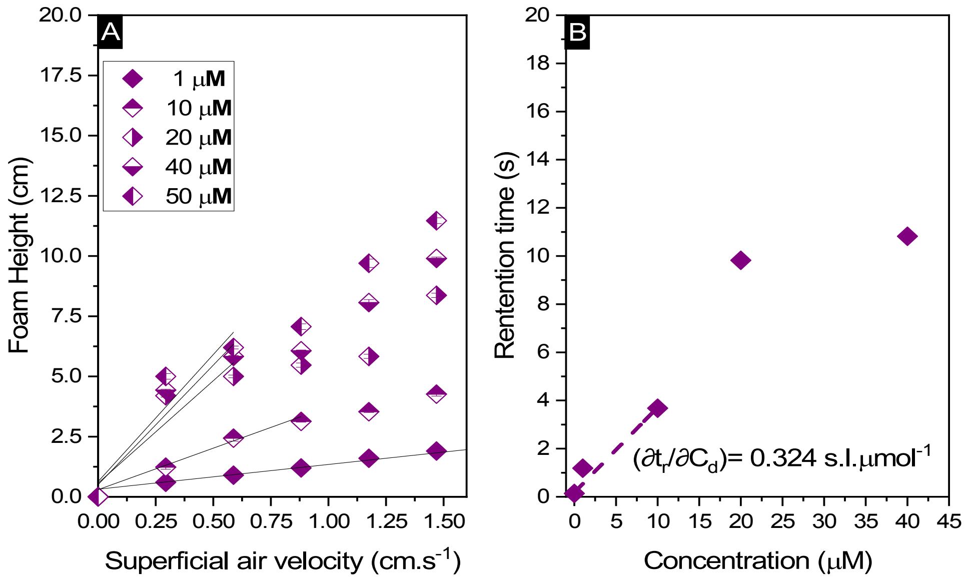

Figure S3: A) The change in foam height with superficial air velocity for sodium lauroyl isethionate without $Mg(OH)_2$ particles. B) The retention time variation (calculated using Eqn. 2) with collector/frother concentration.
